# Revealing Prostate Calcification Heterogeneity through Their Elemental Distribution

**DOI:** 10.1021/cbmi.5c00050

**Published:** 2025-07-07

**Authors:** Sarah B. Gosling, Emily L. Arnold, Lois Adams, Paul Cool, Kalotina Geraki, Mark O. Kitchen, Iain D. Lyburn, Keith D. Rogers, Tim Snow, Nick Stone, Charlene E. Greenwood

**Affiliations:** † School of Chemical and Physical Sciences, 4212 Keele University, Staffordshire, Keele ST5 5BG, U.K.; ‡ Diamond Light Source, Harwell Science and Innovation Campus, Didcot OX11 0DE, U.K.; § 9885 Robert Jones and Agnes Hunt Orthopaedic Hospital NHS Foundation Trust, Shropshire, Oswestry SY10 7AG, U.K.; ∥ School of Medicine, Keele University, Staffordshire, Keele ST5 5BG, U.K.; ⊥ Cranfield Forensic Institute, 151475 Cranfield University, Shrivenham SN6 8LA, U.K.; # Thirlestaine Breast Centre, Gloucestershire Hospitals NHS Foundation Trust, Gloucestershire, Cheltenham GL53 7AS, U.K.; ¶ Cobalt Medical Charity, Cheltenham GL53 7AS, U.K.; ∇ Department of Physics and Astronomy, 3286 University of Exeter, Exeter EX4 4QL, U.K.

**Keywords:** X-ray fluorescence, prostate cancer, calcifications, mapping, biomarkers, synchrotron

## Abstract

Calcifications across the body offer snapshots of the surrounding ionic environment at the time of their formation. Links between prostate calcification chemistry and cancer are becoming of increasing interest, particularly in identifying biomarkers for disease. This study utilizes X-ray fluorescence mapping of 72 human prostate calcifications, measured at the I18 beamline at the Diamond Light Source, to determine the links between calcifications and their environment. This paper offers the first investigation of the elemental heterogeneity of prostate calcifications, demonstrating lower relative levels of minor elements at the calcification center compared to the edge but higher levels of zinc. Importantly, this study uniquely presents links between average Fe, Cr, Mn, Cu, and Ni ratios and grade Group (a classification system for urological tumors, specifically for prostate cancer), highlighting a potential avenue of exploration for biomarkers in prostate calcifications.

## Graphical abstract

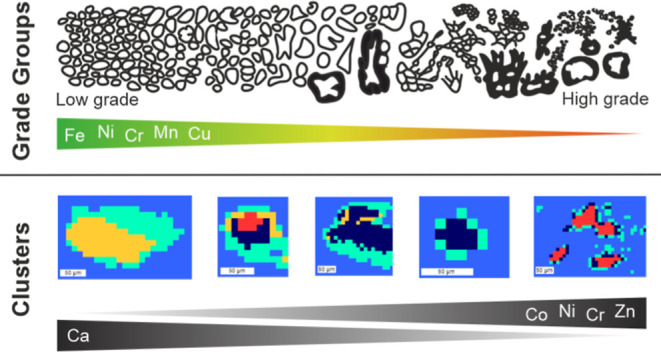

## Introduction

Calcifications (deposits of calcium salts) are found throughout the body associated with benign and malignant conditions, including atherosclerosis, urinary stones, and cancer.^1–6^ The composition of calcifications varies depending on the cause, location, and microenvironment of the deposits. Calcifications are commonly associated with cancer and precancerous disease in the breast, and evidence suggests links between calcification and cancer in other tissues, such as the prostate.^7,8^ Prostate calcifications associated with cancer have recently been investigated using X-ray diffraction, revealing hydroxyapatite (HAP, Ca_10_(PO_4_)_6_(OH)_2_), whitlockite (WH, Ca_9_(MgFe)­(PO_4_)_6_PO_3_OH), and calcium carbonate (CaCO_3_) as key minerals in these deposits, with links between tumor grade and mineral composition and crystallographic structure.^9^

HAP has the propensity to hold multiple ion substitutions, which can impact the crystal structure of the mineral as well as the chemistry. A variety of cations will readily substitute for calcium (Ca) in the HAP crystal lattice, including zinc (Zn), iron (Fe), manganese (Mn), magnesium (Mg), cobalt (Co), and nickel (Ni), with differing effects on crystal properties.^10^ For example, Zn, Mn, or Mg substitution typically destabilizes the HAP lattice, preferentially forming minerals such as WH.^10–12^

Determining the elements present within prostate calcifications may provide key insights into elements involved in the formation and progression of prostate cancer with the HAP and WH lattices acting as ion stores. Previous elemental analyses of prostate calcifications using energy-dispersive X-ray spectroscopy have identified Ca, P, Na, Mg, Al, S, and Zn as key elements, though these were not specifically investigated in the context of prostate cancer.^13,14^

Much of the research into the elemental composition of prostate tissue and its links to prostate cancer formation and progression has focused on soft tissue analysis. Zn is particularly important in prostate cancer and is perhaps one of the most studied elements in this area. Normal prostate tissue has 10–20 times more Zn than normal tissue in other organs, which depletes significantly as tumor grade increases.^15–19^ This is in contrast to breast cancer, where Zn levels increase with malignancy, particularly in triple-positive and triple-negative breast cancer.^20^ Studies in prostate soft tissue also suggest a key role for Fe in prostate cancer progression, where Fe concentrations were found to increase with increasing tumor grade, as measured by Gleason score, which grades cancers based on how abnormal cells appear.^17^ A higher Gleason score is linked to a more aggressive disease.

Levels of other minor elements have also been shown to differ between benign and malignant prostate tissue, including silver (Ag), aluminum (Al), boron (B), bromine (Br), copper (Cu), lithium (Li), Mn, and Ni, with Mn and Cu levels correlating with increasing Gleason score.^17,21^ Similar trends have also been observed in serum, where levels of Zn, Fe, Se, and Mn were found to decrease in prostate cancer patients compared to controls with increased Fe and Zn levels associated with lower recurrence rates in prostate cancer.^22–24^ Therefore, an investigation of these elements in the context of prostate calcifications is essential to understand how different elements are captured and potentially released from the HAP and WH crystal lattices, impacting cancer formation and progression.

Previous studies in breast calcifications have also highlighted individual calcification heterogeneity, with more crystalline HAP in the calcification center compared to the edges, suggesting fewer ion substitutions in the calcification center.^25^ Further, concentric patterns of mineral distribution have been observed, and differential distribution of elements including magnesium at the calcification edge, associated with the presence of WH.^25,26^ Therefore, it is proposed that similar heterogeneity will be observed in prostate calcifications.

In order to analyze this heterogeneity at the micrometer scale, the use of synchrotron techniques is required, facilitating the investigation of features across individual calcifications rather than measuring global averages. This level of spatial resolution is currently achievable only using a microfocus synchrotron source. Synchrotrons also provide high brilliance, allowing measurements to be made much more quickly than on a laboratory source and permitting the collection of large quantities of data.

The aim of this study was to investigate the heterogeneity of individual human prostate calcifications and determine links between elemental ratios and histopathological tissue grade through synchrotron X-ray fluorescence analysis.

## Results and Discussion

### Elemental Composition of Prostate Calcifications

The elemental composition of human prostate calcifications in 10 μm thick FFPE sections was interrogated using X-ray fluorescence. Calcifications contained calcium (Ca), titanium (Ti), chromium (Cr), manganese (Mn), iron (Fe), cobalt (Co), nickel (Ni), copper (Cu), and zinc (Zn), based on the energy range interrogated (Figure 1). All 72 calcifications measured contained Ca, with the majority also containing Zn and Ti (90% and 97% of calcifications, respectively, Table 1). Cr, Mn, Fe, and Co were also present in most calcifications (63%, 56%, 69%, and 74%, respectively), while Ni and Cu were present in a smaller number (42% and 4%, respectively, Table 1). Ca and Zn were the major elements present in all calcifications, making up 98% of the mass fraction of all of the elements measured (Table 1). This is consistent with previous studies which found evidence of Ca and Zn in prostate calcifications and Zn, Ni, and Mn more generally in prostate tissue.^13,14,21^ This also reflects studies in breast calcifications, where Na, Mg, Zn, Fe, Sr, Cu, and Mn were identified associated with HAP calcifications.^27,28^

**Figure 1:**
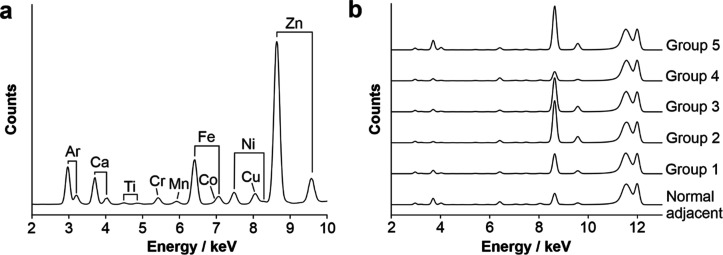
X-ray fluorescence spectra of calcifications. (a) Median spectra from all calcifications measured, with labeled elements. Where two peaks are labeled, these indicate the Kα and Kβ emission lines for each element. For Cr, Mn, Co, and Cu, the Kβ emission lines are masked by other peaks. Co Kα overlaps with Fe Kβ, appearing as a shoulder; however, deconvolution of these peaks shows this is a true signal. More detailed information can be found in Supporting Information Table S1 and Supporting Information Figure S1. (b) Median spectra from each grade Group (1–5) and normal adjacent tissue.

**Table 1:** Overall Distribution of Elements across All Calcifications Measured

element	number of calcifications containing each element (percentage of total/%)	ratio of calcification mass fraction of each element
Ca	72 (100)	0.51
Ti	70 (97)	2.3 × 10^–3^
Cr	45 (63)	5.8 × 10^–4^
Mn	40 (56)	4.0 × 10^–4^
Fe	50 (69)	0.013
Co	53 (74)	1.1 × 10^–3^
Ni	30 (42)	3.5 × 10^–4^
Cu	3 (4)	5.1 × 10^–4^
Zn	65 (90)	0.47

In general, levels of Ca, Zn, and Co were higher in the center of calcifications compared to the edges (Figure 2). There was an average 31-fold increase in Ca in the center compared with the edge, while Zn had a 69-fold increase. Co had a smaller difference between the center and edge of the calcification with only a 13-fold increase. The distribution of these elements within calcifications did not differ among different grade Groups of prostate cancer. Ti, Cr, Mn, and Ni had uniform distributions across the calcifications, while Cu was sparsely distributed (Figure 2).

**Figure 2:**
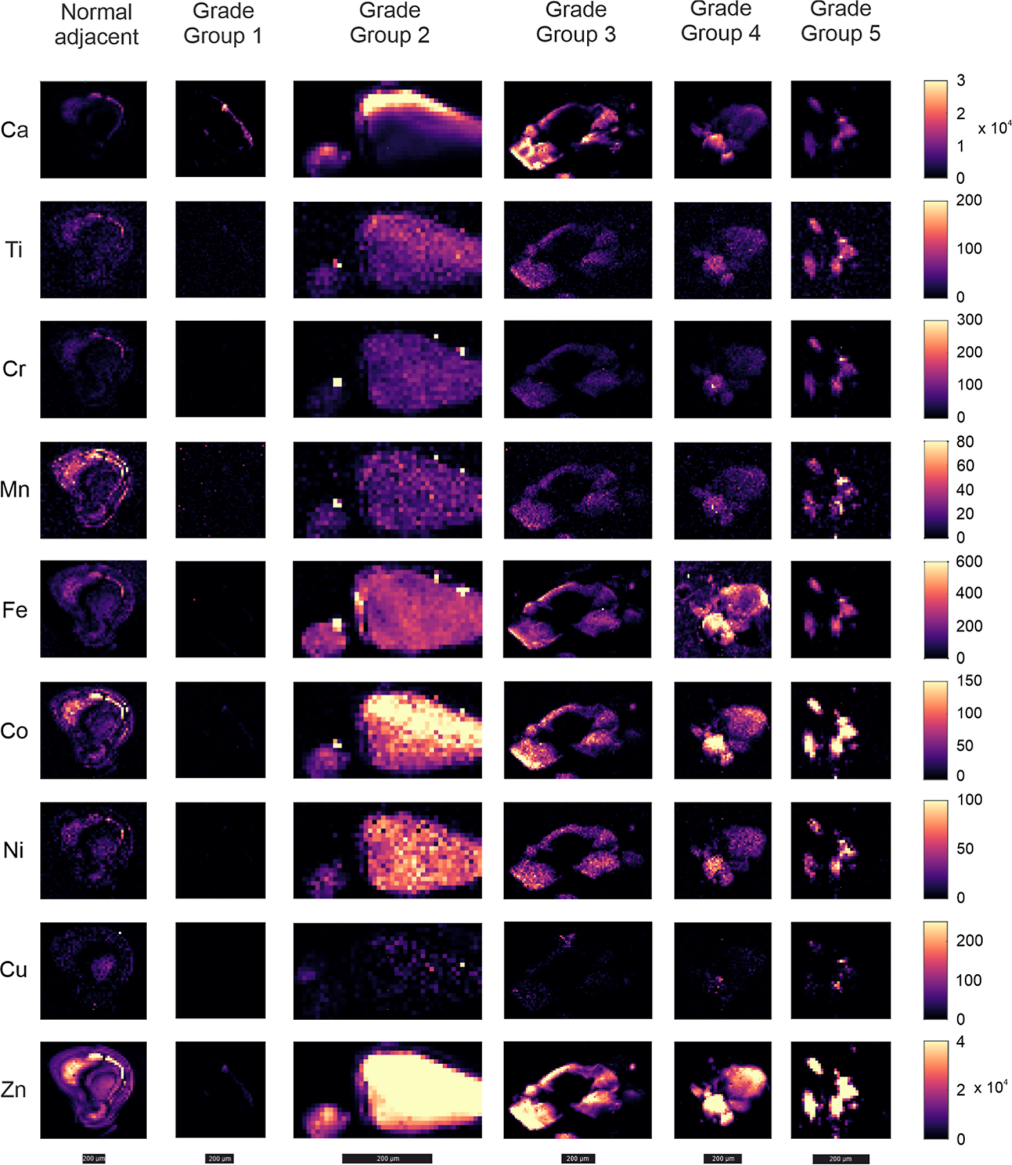
Distributions of elements across example calcifications from different grade Groups. The images have been scaled for ease of interpretation. Each calcification has an individual scale bar at the bottom of each image stack. Scale bar = 200 μm.

### Elemental Ratios Correlate with Grade Group

For many of the elements measured, relative ratios increased from normal adjacent (NA) tissue up to Grade Group 3, ranging from 15% (Fe) to 2098% (Zn) increases. Ratios then decreased to Grade Group 5 (ranging from 20% (Ti and Mn) to 51% (Zn) decreases). The opposite trend was true for the Ca ratio, which decreased 67% between NA and Grade Group 3 and increased 122% to Grade Group 5 (Figure 3). Grade Group 5 had significantly lower levels in relative ratios of Ti, Cr, and Ni compared to Grade Group 3 (20%, 30% and 43% lower, respectively, Figure 3b,c,g), while Grade Group 3 had a significantly higher relative ratio of Cu compared to Grade Group 1 (237% increase, Figure 3h). This is comparative to trends observed by Banas et al. (2010) in prostate soft tissue, where Mn and Cu increased from healthy to Gleason score 2, then decreased to Gleason score 4 ^17^.

**Figure 3:**
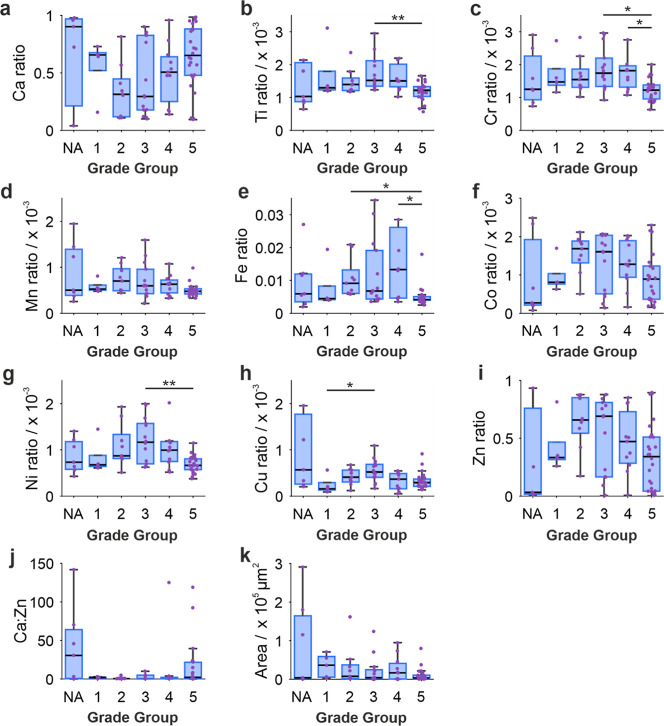
Box charts of each elemental ratio ((a) Ca, (b) Ti, (c) Cr, (d) Mn, (e) Fe, (f) Co, (g) Ni, (h) Cu, (i) Zn, and (j) Ca:Zn) and (k) calcification area, split by Grade Groups (1–5). NA: normal adjacent tissue. *p < 0.05, **p < 0.01, ***p < 0.001 (Kruskal–Wallis). Each data point represents an individual calcification.

Overall, weak negative correlations were observed in Fe, Cr, Mn, Ni, and Cu ratios with Grade Group (Supporting Information Table S2). Other studies investigating prostate soft tissue, rather than calcifications, have found the opposite trend, where a higher level of these elements is associated with prostate cancer or increasing Grade Group.^17,21,29^ Similar studies in breast calcifications have highlighted higher levels of Fe in malignant calcifications compared to benign, demonstrating the opposite trend to that presented here for Fe, perhaps indicating a differential role for Fe in breast and prostate cancers.^28,30^ However, these studies did not separate malignant tissue by grade; therefore, this could reveal different overall trends.

Metal ion substitution into the HAP and WH lattices has been shown to have differential effects of the solubility of these minerals.^31^ Fe, Ni, Mn, Cr, and Cu can increase the solubility of HAP in physiological conditions, causing a higher rate of mineral dissolution and release of ions into the tissue microenvironment.^10,32–35^ Therefore, the presence of these minerals in the HAP or WH lattices can impact the bioactivity of calcifications. Release of these ions at lower grades may facilitate the early progression of cancer due to the biological functions of these ions.

Fe plays a key role in tumor proliferation through the modulation of enzymes linked to androgen receptor (AR) expression and activity. For example, Fe^2+^ destabilizes hypoxia inducible factor 1 alpha (HIF1α), which can lead to the upregulation of AR expression.^36^ Increased AR expression subsequently leads to the promotion of tumor growth. Therefore, the release of Fe into the soft tissue from calcifications could promote tumor proliferation. Similarly, Ni interacts with HIF-1α to induce hypoxic effects and promote tumorigenesis in prostate cancer.^37,38^ Further, Cr has been reported to promote mesenchymal markers and downregulate epithelial markers, facilitating the epithelial–mesenchymal transition and promoting cell invasion.^37,39^ Conversely, Mn has been shown to have anticancer effects, reducing the cell viability of prostate cancer cell lines by inducing cell cycle arrest and apoptosis.^40^ Therefore, the release of these minor elements into the tumor microenvironment may have both protective and carcinogenic effects.

No correlations were observed in Ca, Zn, Co, or Ti ratios with Grade Group. This is in agreement with previous studies into prostate soft tissue, which also found no correlation between tissue grade and levels of Co or Ti. However, earlier reports have shown negative correlations between Ca and Zn levels and soft tissue pathology, which contrasts with the findings presented here.^17,21,29^

This indicates that the elemental distribution in calcifications differs from that in soft tissue. This is perhaps an expected finding, given the ability of HAP and WH crystal lattices to be readily substituted by other ions, particularly in biological systems.^41,42^ For example, carbonate is often readily substituted for phosphate in biological tissues, and ions such as Fe or Na substituting for Ca in HAP, or Mg substituting for Ca in WH.^12,41–43^ This may suggest that calcifications act as an ion store for minor elements at lower grades, which can be released into the surrounding tissue as cancer progresses, causing higher levels of these ions in the soft tissue. This likely occurs through the reabsorption of calcifications and the release of ions into the surrounding microenvironment. This would, in turn, cause changes in the levels of these ions in the soft tissue.

### Cluster Analysis of Elemental Maps

Elemental maps were decomposed using a k-means clustering approach. Cluster 1 represented the surrounding soft tissue, while clusters 2–5 were all contained within the calcification. The remainder of this analysis will focus on clusters 2–5.

Where all four clusters were present (6 calcifications), clusters were ordered, from the calcification center to the edge 2 > 4 > 5 > 3. Clusters 2 and 4 had the lowest Ca ratios (0.13 and 0.18, respectively) and highest Zn ratios (0.85 and 0.81, respectively) (Figure 4a,i, Supporting Information Table S3). Cluster 5 was found toward the center of small calcifications and toward the edge of larger calcifications and had the lowest Zn ratio (0.21) and highest Ca ratio (0.75). Cluster 3 had a lower Zn ratio (0.74) and a higher Ca ratio (0.23). Higher Fe ratios were observed in clusters 3 and 4 (1.03 × 10^–2^ and 6.61 × 10^–3^, respectively, Figure 4e) while these were lower in clusters 2 and 5 (4.37 × 10^–3^ and 4.04 × 10^–3^, respectively). Similar trends were observed for Ti, Cr, Ni, Mn, Co, and Cu (Figure 4, Supporting Information Table S3).

**Figure 4:**
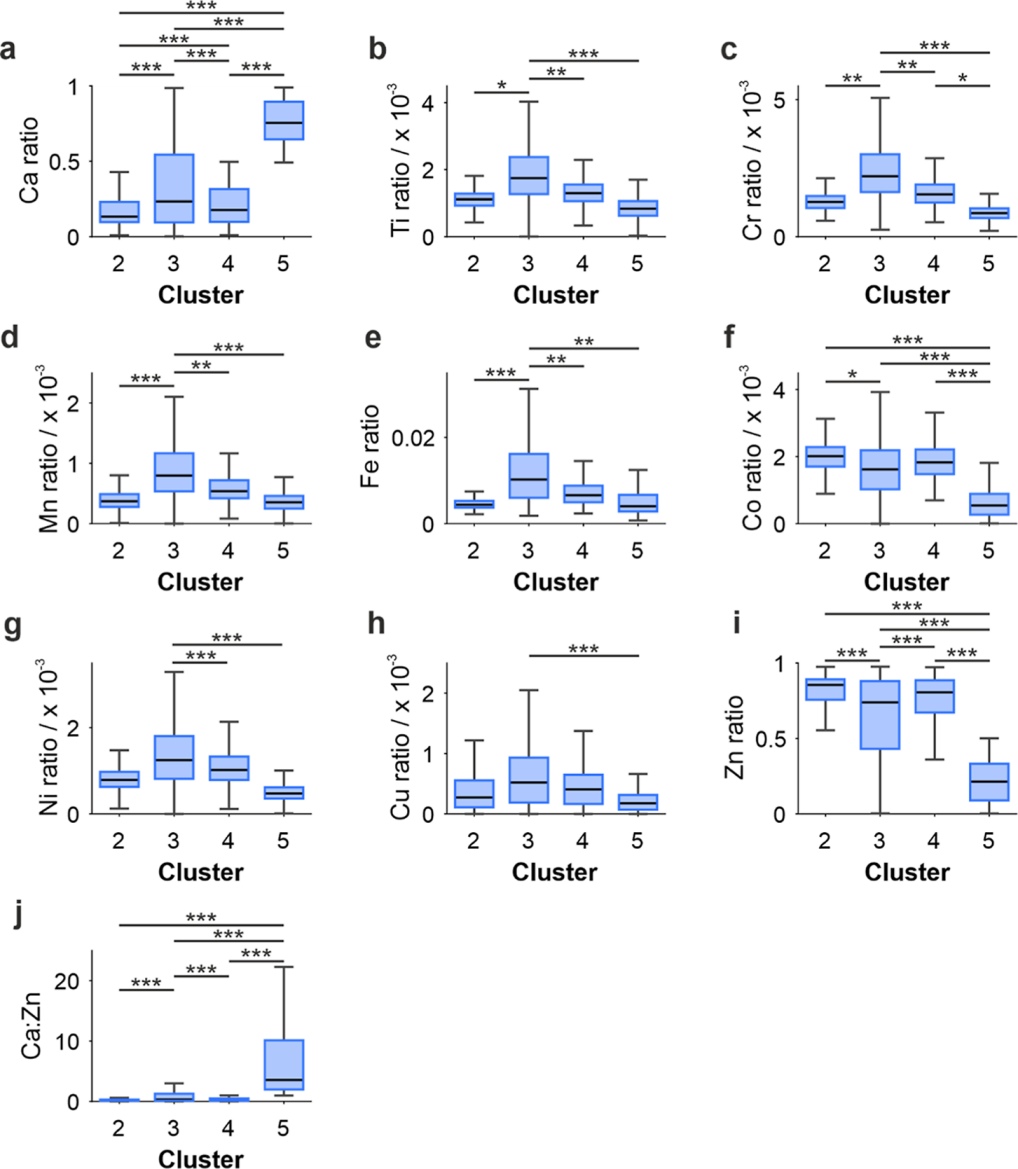
Box charts of each elemental ratio ((a) Ca, (b) Ti, (c) Cr, (d) Mn, (e) Fe, (f) Co, (g) Ni, (h) Cu, (i) Zn, and (j) Ca:Zn) split by clusters (2–5). Cluster 1 is not included as this is not a calcification cluster. *p < 0.05, **p < 0.01, ***p < 0.001 (Kruskal–Wallis). Individual data points are not visualized for simplicity (*n* > 100,000).

This demonstrates a higher Zn ratio in the center of calcifications, with all other elements having a lower ratio toward the calcification center. This may suggest that Zn-substituted HAP is the predominant mineral in these calcifications, which has also been suggested in *in vitro* osteoblast experiments.^44^ However, Zn substitution into the HAP lattice typically has a destabilizing effect, leading to the preferential formation of minerals such as WH.^10–12^ Previous XRD studies have shown no statistical differences in the level of WH in prostate calcifications between normal adjacent and malignant tissue calcifications.^9^ Therefore, Zn may be present as a labile ion, rather than being incorporated directly into the lattice, as suggested elsewhere.^45^ A lower level of minor elements may also suggest that minerals in the center of calcifications are more mature, having lower levels of substitution within the HAP lattice, consistent with findings in other studies investigating calcifications in the breast.^25^

### Grouping Calcifications by Type

Calcifications were subsequently grouped into five types based on the distribution of clusters 2–5 in the false color maps. Type A contained clusters 3 and 5; type B contained all four calcification clusters; type C contained clusters 3, 4, and 5; type D contained clusters 3 and 4; type E predominantly consisted of clusters 2, 3, and 4 (Figure 5).

**Figure 5:**
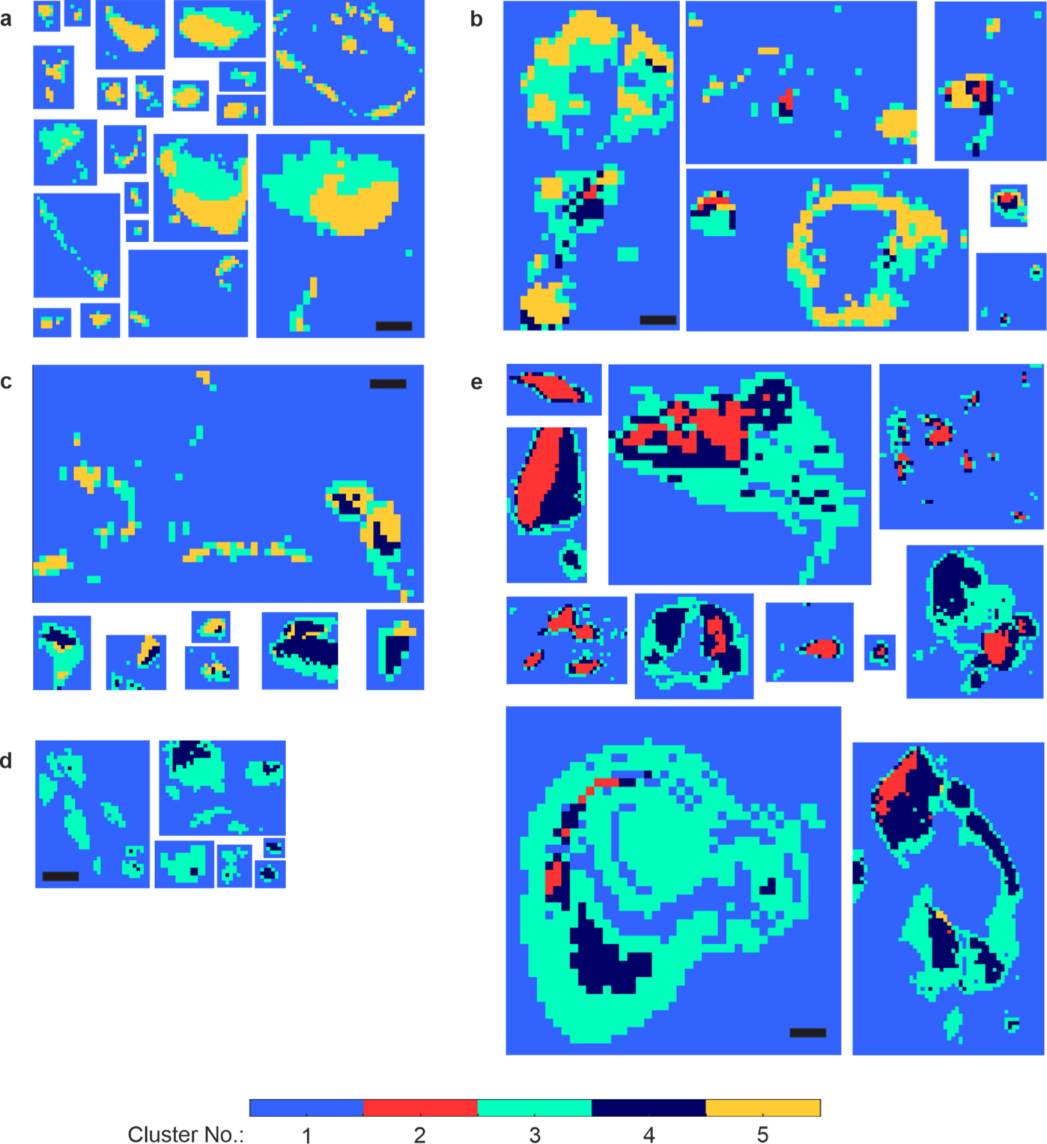
Calcification types (A–E) based on cluster false color maps. All calcifications of the same type are scaled to one another for easy visualization. Scale bar = 100 μm.

Types B and D contained a higher proportion of lower Grade Groups and normal adjacent tissue (67 and 75% of calcifications below Grade Group 3, respectively), while types A and C contained a higher proportion of higher Grade Groups (4–5) (73% and 71%, respectively) (Figure 6a). Type E calcifications contained an equal distribution of low and high Grade Groups. However, there was a distribution of all Grade Groups across calcification types A to E, meaning that calcification type based on elemental composition did not directly correlate with Grade Group. Therefore, other potential similarities between groups were investigated.

**Figure 6:**
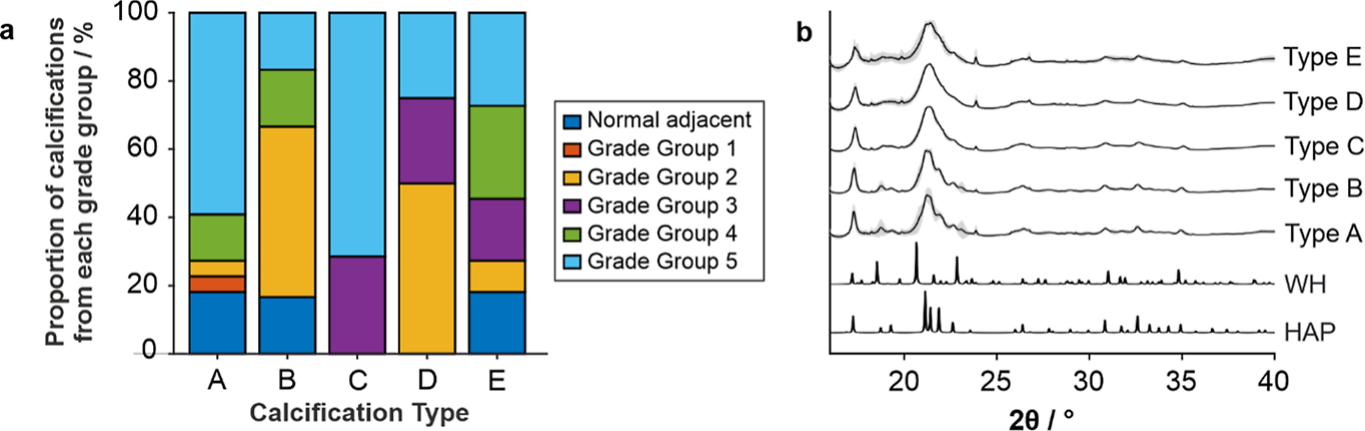
(a) Distribution of Grade Groups of calcifications of each type (A–E) and (b) average (mean) diffractograms for each type with standard deviations in gray and standard diffractograms of hydroxyapatite (HAP) and whitlockite (WH).

Comparing the average X-ray diffraction patterns for each calcification type, type A and type B calcifications appear to contain more crystalline HAP, while types C, D, and E are less crystalline. There is also evidence of WH in type A and type B calcifications, while this is not evident in the other calcification types (Figure 6b). Diffraction data do not appear to link directly to the calcification types, with no correlations between elemental ratios and HAP crystallinity or presence of WH (Figure 7a–j). As discussed above and elsewhere,^10,26,43^ multiple elements can impact the presence of minerals such as WH, including Zn, Mn, and Mg, which cause preferential formation of WH and stabilize the structure. Further, Zn, Fe, and Cu can decrease the crystallinity of HAP, while Cr, Co, and Ni increase this. As noted above, Zn (and other ions) may be present as a labile ion, which may not directly impact HAP crystallinity, explaining the lack of correlation between elemental ratios and crystallographic properties.^45^ Therefore, further investigation is required to directly compare diffraction and fluorescence data of prostate calcifications.

**Figure 7:**
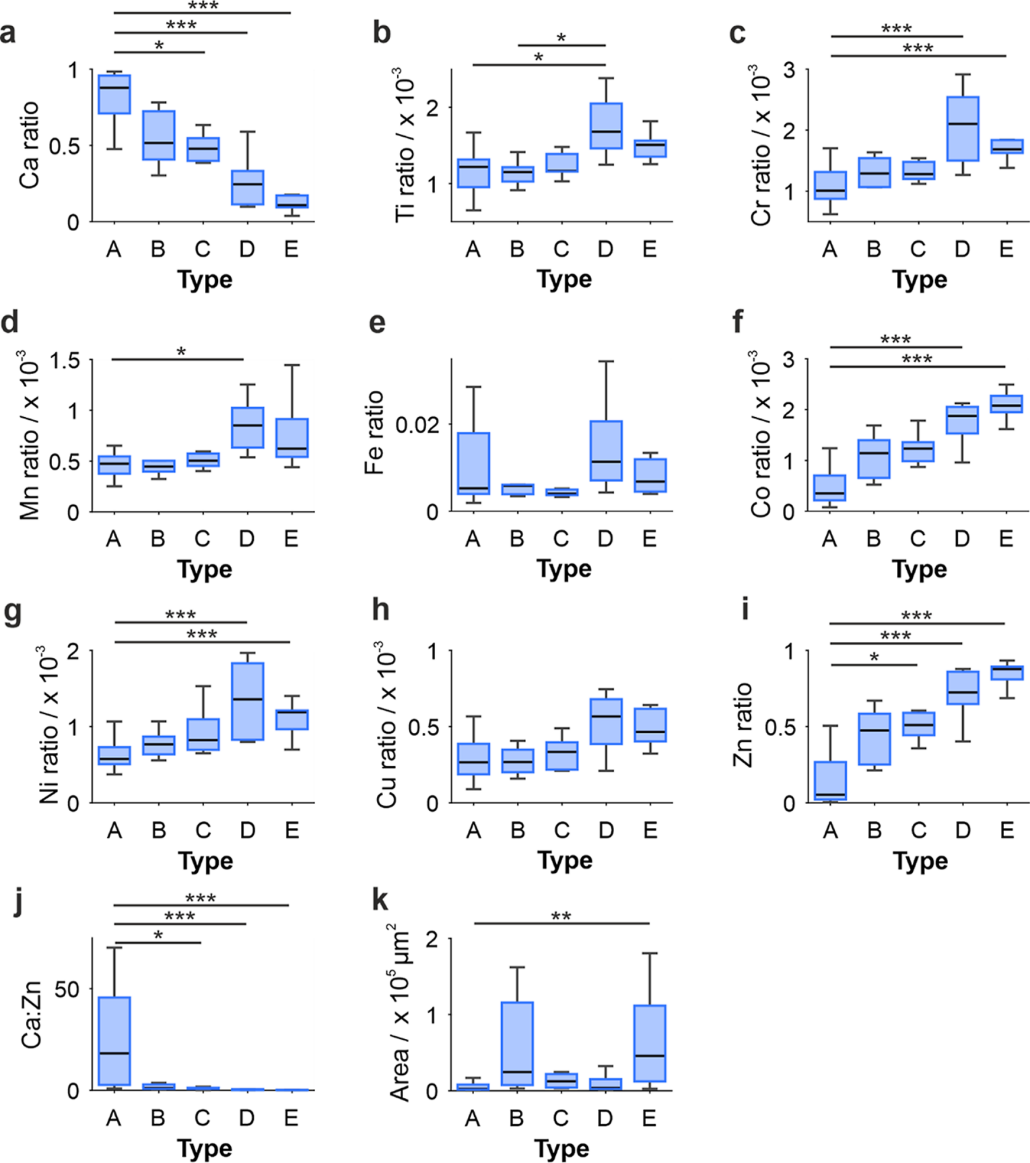
Box charts of each elemental ratio ((a) Ca, (b) Ti, (c) Cr, (d) Mn, (e) Fe, (f) Co, (g) Ni, (h) Cu, (i) Zn, and (j) Ca:Zn) and (k) calcification area split by calcification types (A–E). *p < 0.05, **p < 0.01, ***p < 0.001 (Kruskal–Wallis). Individual data points are not visualized for simplicity.

### Calcification Size and Elemental Ratios

There was a moderate positive correlation (*r*_τ_ = 0.40, *p* = 7.52 × 10^–6^) between the calcification type and calcification area, suggesting that elemental composition may be affected by the size of calcifications. Type A calcifications were the smallest (2500 μm^2^), increasing A < D < C < B < E to type E (45,800 μm^2^) (Figure 7k, Supporting Information Table S4). It is important to note that the areas calculated are from 10 μm thick sections and therefore may not be reflective of the true size or shape of calcifications, as they only reflect a random cross-section of the whole calcification.

There were also weak positive correlations between calcification size and Zn ratio, Cr ratio, Co ratio, and Ni ratio and a weak negative correlation between Ca ratio and calcification size (Figure 7, Supporting Information Table S4), suggesting that larger calcifications contained higher relative levels of Zn, Cr, Co, and Ni compared to smaller calcifications.

## Limitations

This study utilized synchrotron techniques in order to investigate prostate calcifications at high resolution and map the elemental distribution across individual calcium deposits. However, employing microfocus (10 μm spot and step size) mapping methodologies and time constraints at synchrotron facilities leads to relatively small sample numbers but with high levels of detail. This study has provided an initial assessment of the elemental composition of human prostate calcifications, and further work will include the analysis of larger sample sets and investigation of the use of high-throughput techniques to investigate the potential of this work as a prognostic tool.

Further, tissue fixation and embedding protocols have the potential to impact calcification chemistry due to the alteration of the pH and ionic environment of the samples. However, buffering formalin to a neutral pH, which is common practice in the fixation and embedding procedures, negates this effect due to the shift in equilibrium toward the mineralized state of HAP. Formalin fixation of hard tissues has been found to have no impact on crystallographic and elemental properties of HAP, including Ca:P ratio and Ca, Zn, and Fe levels, likely due to binding of these elements into the lattice.^46,47^ Conversely, labile elements have greater potential for redistribution or enhanced binding during the fixation process. Further, studies have demonstrated that transition metals (Ti, Cr, Mn, Fe, Co, Ni, Cu, and Zn) are less affected by the fixation and embedding process than alkali metals.^48^ To validate these results, future analysis of fresh frozen tissue samples is currently being pursued by the authors. However, due to current biobank procedures, which involve the fixation of prostate tissue, this may further reduce the number of samples available for analysis.

## Conclusion

This paper has, for the first time, demonstrated the heterogeneity of elemental distributions within and between prostate calcifications through synchrotron X-ray fluorescence mapping. Correlations between the calcification area and elemental ratios have been highlighted, suggesting that the calcification size may be linked to the elemental composition.

Importantly, for developing new biomarkers for prostate cancer, relative ratios of Fe, Cr, Cu, Mn, and Ni have been directly correlated with the tissue Grade Group. These ions play key roles in cancer formation and progression through acting as enzyme cofactors or disrupting cell signaling pathways involved in disruption of the extracellular matrix, the epithelial–mesenchymal transition, and cell migration.^15,36,49^ Therefore, prostate calcifications may play a role in ion storage, facilitating these processes.

Further investigation of lighter elements, including Mg and Na, is essential to develop a comprehensive understanding of the elemental composition of calcifications and how this might link to the surrounding tissue microenvironment and disease.

## Materials and Methods

### Samples

All samples were from radical prostatectomy surgeries, sourced from the Human Biomaterials Research Centre (HBRC) in Birmingham, with ethical approval received through NHS REC (25/NW/0013). Formalin-fixed paraffin-embedded (FFPE) sections of 10 μm thickness were taken from prostate megablocks using a microtome including a range of Grade Groups (1–5) and from matched normal adjacent tissue. Grade Groups are defined according to the International Society of Urological Pathology (ISUP) Grade (Groups 1–5), which is a classification system for urological tumors, specifically for prostate cancer.^50^ Grade 1 tumors are slow growing and the least aggressive, while Grade 5 tumors are fast growing and the most aggressive. Normal adjacent tissue was taken from patients with Grade Group 2 (2 patients) and Grade Group 5 (2 patients) prostate tumors due to availability and was determined to be normal via histopathology. 72 individual calcifications across 49 sections were selected for analysis (7× normal adjacent, 5× Grade Group 1, 9× Grade Group 2, 14× Grade Group 3, 11× Grade Group 4, and 26× Grade Group 5).

### Synchrotron X-ray Analysis

X-ray fluorescence and diffraction data were collected consecutively on the I18 beamline at Diamond Light Source, Didcot, UK, with a beam energy of 12 keV and a probe spot size of 10 μm × 10 μm. Maps were collected using a 10 or 20 μm step size. Samples were mounted perpendicular to the X-ray beam and a microscope mounted at 45° to the sample stage was used to identify regions of interest using corresponding microcomputed tomography (μCT) section images as a reference (Supporting Information Figure S2).

## X-ray Fluorescence

X-ray fluorescence maps were collected using a 10 or 20 μm step size with each point collected for 0.1 s per point using a Vortex silicon drift detector, mounted at 90° to the X-ray beam. Data were normalized to the Rayleigh elastic scatter peak at 12 keV, and elemental assignment and peak intensities of argon, calcium, titanium, chromium, manganese, iron, cobalt, nickel, and zinc were measured using PyMCA 5.9.4 ^51^ and mass fraction values calculated. This study did not utilize a standard to accurately calculate absolute concentrations of elements; therefore, all presented values are ratios of each element mass fraction relative to the total mass fraction of all elements measured. For example, one sample had a Ca mass fraction of 8.5 × 10^–7^ and Zn mass fraction of 1.4 × 10^–6^, with a total mass fraction of 3.9 × 10^–6^, therefore, these are reported as 0.22 and 0.36.

It was not possible to detect elements below Ar using the methodology in this paper, therefore, the elemental composition presented here does not include elements such as P, S, Mg, Na, Al, and Cl, which have been implicated elsewhere.^13,14,43^ However, these elements were not the focus of this work.

### X-ray Diffraction

X-ray diffraction data were collected for 15 s per point using an Excalibur detector, using a 10 μm step size. Data were calibrated using a silicon standard and a detector mask applied, before azimuthally integrating 2-dimensional data into 1-dimensional data using the Diamond Analysis WorkbeNch (DAWN) software (V2.26.0, Diamond Light Source).^52^ Phases were identified using the International Centre for Diffraction Data (ICDD) database (PDF-5+, 2023) and JADE Pro (ICDD).

### Cluster Analysis and Mapping

Relative quantification of elemental levels was decomposed by a k-means clustering approach. The optimal number of clusters was determined using the elbow method (Supporting Information Figure S3). Separation of clusters 1 and 2 was highly affected by the surrounding soft tissue, initially suggesting that two clusters may be the optimal number. However, in order to also cluster within calcifications, k must be greater than 2, therefore, *k* > 2 was also explored using the elbow method, resulting in the use of five clusters for the full analysis. Five clusters and a squared Euclidean distance metric (where the centroid is the mean of the points in each cluster) were used to decompose the calcification data, with a maximum number of iterations of 100.^53,54^ Data were visualized using false color maps generated in MATLAB 2023a (The MathWorks Inc., Natick, Massachusetts). Calcifications were separated into five groups (A–E) based on the distribution of clusters within calcifications (Table 2) and median values were calculated for each group.

**Table 2:** Distribution of Calcification Clusters within Each Calcification Type

	clusters within each calcification type			
calcification type	2	3	4	5
A		√		√
B	√	√	√	√
C		√	√	√
D		√	√	
E	√	√	√	

### Statistics

All included parameters failed a Shapiro–Wilk test for normality; therefore, median values were calculated and reported alongside interquartile ranges. Groups were compared using Kruskal–Wallis tests, with a Dunn–Sidak posthoc correction for multiple comparisons. All quoted *p* values are Dunn–Sidak-corrected values unless otherwise stated.

To assess the correlation between continuous and ordinal parameters, Kendall’s tau correlation coefficients (*r*_τ_) were calculated and reported alongside corresponding *p* values. The sample numbers and nonparametric nature of this data deemed Kendall’s tau the most appropriate statistical test.

All mathematical analyses were performed using MATLAB 2023a (The MathWorks Inc., Natick, Massachusetts).

## Supplementary Material



## Notes

Details about elemental fitting, statistical and *p* values for Kendall’s tau and Kruskal–Wallis, elemental ratios for clusters, and example CT images and elbow curves for *k*-means clustering (PDF)
