# Supplementary material for: Revealing Prostate Calcification Heterogeneity through Their Elemental Distribution
Source: Chem Biomed Imaging. 2025 Jul 7;3(12):865–75. doi: 10.1021/cbmi.5c00050 (PMC12728748; doi:10.1021/cbmi.5c00050)
Supplement: Supplementary file 1 [file im5c00050_si_001.pdf]

Supplementary Information

Revealing prostate calcification heterogeneity through their elemental distribution

Sarah B. Gosling[a,], Emily L. Arnold[b], Lois Adams[a], Paul Cool[c,d], Kalotina Geraki[b], Mark O. Kitchen[d], Iain D. Lyburn[e,f,g], Keith D. Rogers[e], Tim Snow[b], Nick Stone[h] & Charlene E. Greenwood[a,*].*

a. School of Chemical and Physical Sciences, Keele University, Keele, Staffordshire, ST5 5BG, UK

b. Diamond Light Source, Harwell Science and Innovation Campus, Didcot, OX11 0DE, UK

c. Robert Jones and Agnes Hunt Orthopaedic Hospital NHS Foundation Trust, Oswestry, Shropshire, SY10 7AG, UK

d. School of Medicine, Keele University, Keele, Staffordshire, ST5 5BG, UK

e. Cranfield Forensic Institute, Cranfield University, Shrivenham, SN6 8LA, UK

f. Thirlestaine Breast Centre, Gloucestershire Hospitals NHS Foundation Trust, Cheltenham, Gloucestershire, GL53 7AS, UK

g. Cobalt Medical Charity, Cheltenham, GL53 7AS, UK

h. Department of Physics and Astronomy, University of Exeter, Exeter, EX4 4QL, UK

*Emails: Sarah Gosling – s.b.gosling@keele.ac.uk,

Charlene Greenwood – c.e.greenwood@keele.ac.uk

Contents

Title	Content	Page
Table S1	Energies of K-shell emission lines, in kilo electron volts (keV), for elements fitted to calcification data, from X-Ray Data Booklet[1]	3
Figure S1	X-ray fluorescence spectra of calcifications and fit from PyMCA	4
Table S2	Elemental ratios for each Grade Group with Kendall's tau (r_τ) statistics and related *p* values.	5
Table S3	*p* values for comparison of calcifications from normal adjacent tissue (NA) and Grade Groups 1 – 5 using Kruskal-Wallis tests, with a Dunn-Sidak post-hoc correction for multiple comparisons.	6
Table S4	Elemental ratios for each calcification cluster.	7
Table S5	*p* values for comparison of calcification clusters (2 – 4) using Kruskal-Wallis tests, with a Dunn-Sidak post-hoc correction for multiple comparisons.	8
Table S6	Median calcification area and elemental ratios for each cluster type (A – E), with Kendall's tau (r_τ) statistics and related *p* values for each parameter.	9
Table S7	*p* values for comparison of calcification types (A – E) using Kruskal-Wallis tests, with a Dunn-Sidak post-hoc correction for multiple comparisons.	10
Figure S2	Example µCT images of prostate megablocks from each Grade Group	11
Figure S3	Elbow curve using sum of squared distances plot for k = 1 to k = 10 clusters, with optimal number of clusters.	12

Supplementary Table S1. Energies of K-shell emission lines, in kilo electron volts (keV), for elements fitted to calcification data, from X-Ray Data Booklet[1]

**Element**	**Kα**	**Kβ**
Ar	2.96	3.19
Ca	3.69	4.01
Ti	4.51	4.93
Cr	5.41	5.95
Mn	5.90	6.49
Fe	6.40	7.06
Co	6.93	7.65
Ni	7.48	8.26
Cu	8.05	8.91
Zn	8.64	9.57

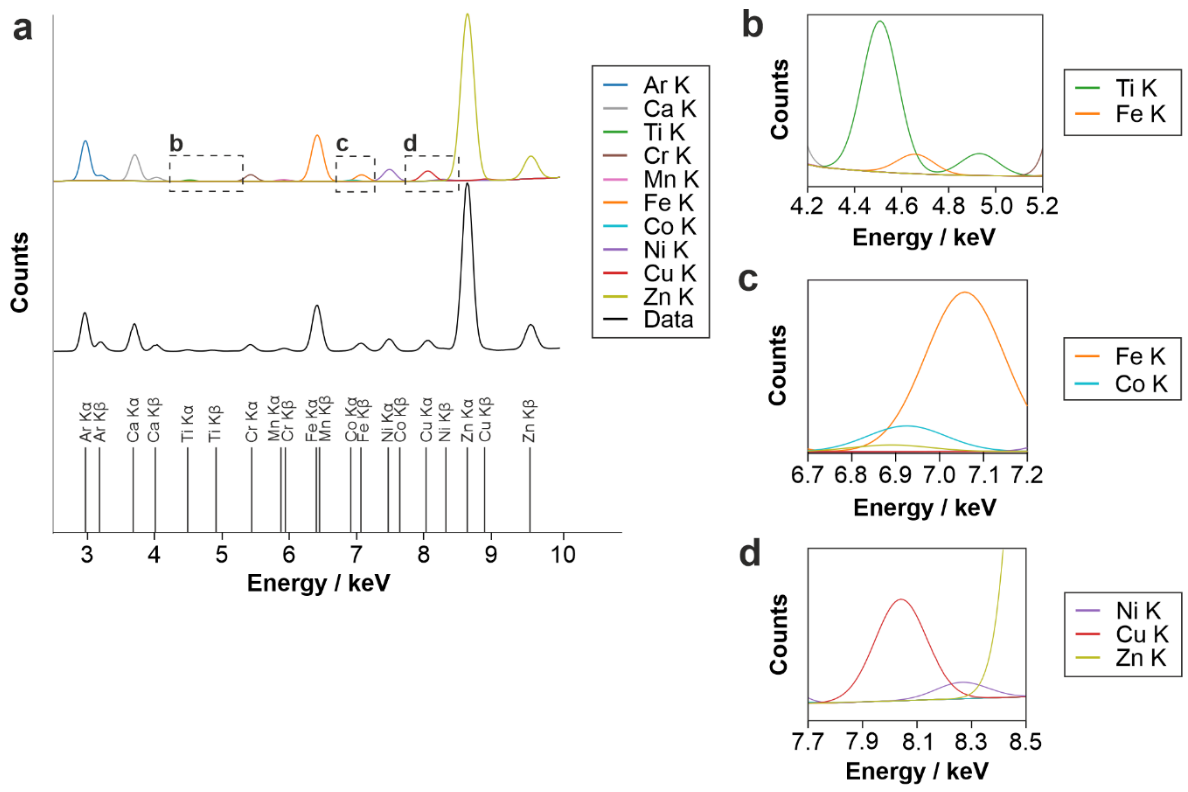

Supplementary Figure S1. X-ray fluorescence spectra of calcifications and fit from PyMCA. a) Median spectra from all calcifications measured, with fits for each element and labeled emission lines. b) Inset of fitted spectra in panel (a) between 4.2 and 5.2 keV, highlighting Ti Kα and Kβ peaks. c) Inset of fitted spectra in panel (a) between 6.7 and 7.2 keV, highlighting Fe Kβ and Co Kα peaks. d) Inset of fitted spectra in panel (a) between 7.7 and 8.5 keV, highlighting Cu Kα and Ni Kβ peaks.

Supplementary Table S2. Elemental ratios for each Grade Group with Kendall's tau (r_τ) statistics and related *p* values. Median values are presented with interquartile ranges in brackets.

	Normal adjacent	Grade Group					r_τ	p
		1	2	3	4	5		
Calc Area /μm^2	3800 (2575 – 164200)	36400 (5300 – 59100)	7500 (1625 – 37275)	4000 (2600 – 24700)	16600 (525 – 41250)	3550 (1900 – 10600)	-0.169	0.055
Ca	0.90 (0.21 – 0.97)	0.66 (0.52 – 0.68)	0.31 (0.12 – 0.45)	0.29 (0.18 – 0.83)	0.51 (0.25 – 0.64)	0.65 (0.48 – 0.88)	0.133	0.199
Ti (x 10^{-3})	1.03 (0.87 – 2.06)	1.30 (1.23 – 1.80)	1.40 (1.21 – 1.59)	1.53 (1.34 – 2.12)	1.51 (1.33 – 2.02)	1.22 (1.03 – 1.33)	-0.156	0.133
Cr (x 10^{-3})	1.25 (0.93 – 2.27)	1.47 (1.37 – 1.87)	1.54 (1.31 – 1.87)	1.74 (1.34 – 2.20)	1.82 (1.31 – 1.97)	1.22 (0.96 – 1.38)	-0.221	**0.033**
Mn (x 10^{-4})	4.99 (3.88 – 13.9)	5.22 (5.02 – 6.11)	6.97 (4.90 – 9.66)	5.93 (4.26 – 9.56)	6.28 (4.42 – 7.22)	4.75 (4.19 – 5.32)	-0.240	**0.020**
Fe (x 10^{-3})	5.88 (3.47 – 11.9)	4.41 (4.12 – 8.25)	9.06 (6.05 – 13.2)	6.75 (4.33 – 19.1)	13.3 (4.60 – 26.2)	4.08 (3.97 – 5.06)	-0.277	**0.007**
Co (x 10^{-3})	0.27 (0.21 – 1.92)	0.81 (0.76 – 1.04)	1.69 (1.31 – 1.89)	1.61 (0.51 – 2.04)	1.28 (0.94 – 1.90)	0.89 (0.37 – 1.23)	-0.131	0.205
Ni (x 10^{-4})	7.31 (5.79 – 11.8)	6.75 (6.39 – 8.82)	8.69 (8.13 – 13.3)	11.6 (6.97 – 15.7)	9.91 (7.50 – 11.8)	6.62 (5.73 – 8.06)	-0.276	**0.008**
Cu (x 10^{-4})	5.67 (2.60 – 17.7)	1.54 (1.34 – 2.92)	4.08 (3.05 – 5.63)	5.19 (4.07 – 6.88)	3.67 (1.59 – 4.89)	2.90 (2.10 – 3.72)	-0.287	**0.005**
Zn	0.03 (0.02 – 0.76)	0.33 (0.31 – 0.47)	0.66 (0.54 – 0.85)	0.69 (0.17 – 0.81)	0.47 (0.28 – 0.73)	0.34 (0.04 – 0.51)	-0.120	0.247
Ca:Zn	30.5 (0.75 – 64.1)	1.97 (1.43 – 2.19)	0.48 (0.14 – 0.86)	0.43 (0.22 – 4.99)	1.07 (0.40 – 2.15)	1.92 (0.94 – 21.81)	0.130	0.211

Significant values ($p<0.05$) are highlighted in **bold**.

Supplementary Table S3. Comparison of calcifications from normal adjacent tissue (NA) and Grade Groups 1 – 5 using Kruskal-Wallis tests, with a Dunn-Sidak post-hoc correction for multiple comparisons. All quoted *p* values are Dunn-Sidak corrected values unless otherwise stated. All significant values are highlighted (Red: $p<0.05$, Yellow: $p<0.01$, Green: $p<0.001$).

Ca ratio

	NA	GG1	GG2	GG3	GG4
GG1	0.993				
GG2	0.270	0.766			
GG3	0.584	0.966	0.974		
GG4	0.831	0.997	0.886	0.999	
GG5	1.000	0.999	0.143	0.422	0.805

Ti ratio

	NA	GG1	GG2	GG3	GG4
GG1	0.997				
GG2	0.981	1.000			
GG3	0.392	0.855	0.818		
GG4	0.676	0.967	0.968	0.998	
GG5	0.989	0.869	0.584	0.006	0.068

Cr ratio

	NA	GG1	GG2	GG3	GG4
GG1	0.983				
GG2	0.936	1.000			
GG3	0.599	0.988	0.989		
GG4	0.732	0.996	0.998	1.000	
GG5	0.955	0.607	0.247	0.011	0.044

Mn ratio

	NA	GG1	GG2	GG3	GG4
GG1	1.000				
GG2	0.992	0.997			
GG3	1.000	1.000	0.999		
GG4	1.000	1.000	0.996	1.000	
GG5	0.869	0.895	0.336	0.445	0.646

Fe ratio

	NA	GG1	GG2	GG3	GG4
GG1	0.997				
GG2	0.798	0.575			
GG3	0.987	0.885	0.967		
GG4	0.843	0.622	1.000	0.984	
GG5	0.768	0.990	0.022	0.094	0.019

Co ratio

	NA	GG1	GG2	GG3	GG4
GG1	0.999				
GG2	0.338	0.709			
GG3	0.699	0.953	0.967		
GG4	0.736	0.959	0.974	1.000	
GG5	1.000	1.000	0.200	0.576	0.660

Ni ratio

	NA	GG1	GG2	GG3	GG4
GG1	1.000				
GG2	0.833	0.730			
GG3	0.642	0.540	1.000		
GG4	0.939	0.862	0.999	0.987	
GG5	0.908	0.994	0.073	0.007	0.133

Cu ratio

	NA	GG1	GG2	GG3	GG4
GG1	0.109				
GG2	0.982	0.316			
GG3	1.000	0.043	0.954		
GG4	0.438	0.874	0.839	0.213	
GG5	0.263	0.842	0.696	0.056	1.000

Zn ratio

	NA	GG1	GG2	GG3	GG4
GG1	0.991				
GG2	0.304	0.818			
GG3	0.671	0.987	0.963		
GG4	0.908	1.000	0.832	0.997	
GG5	1.000	0.998	0.175	0.547	0.914

Ca:Zn ratio

	NA	GG1	GG2	GG3	GG4
GG1	0.991				
GG2	0.293	0.808			
GG3	0.666	0.987	0.960		
GG4	0.902	1.000	0.829	0.997	
GG5	1.000	0.997	0.156	0.522	0.896

Calcification Area

	NA	GG1	GG2	GG3	GG4
GG1	0.996				
GG2	0.999	0.960			
GG3	0.985	0.848	0.999		
GG4	0.964	0.787	0.996	1.000	
GG5	0.808	0.521	0.931	0.984	0.999

Supplementary Table S4. Elemental ratios for each calcification cluster. Median values are presented with interquartile ranges in brackets.

	**Cluster**			
	**2**	**3**	**4**	**5**
Ca	0.13 (0.10 – 0.23)	0.23 (0.10 – 0.54)	0.18 (0.10 – 0.32)	0.75 (0.64 – 0.90)
Ti ($x 10^{-3}$)	1.11 (0.93 – 1.28)	1.74 (1.27 – 2.38)	1.30 (1.06 – 1.55)	0.84 (0.63 – 1.06)
Cr ($x 10^{-3}$)	1.27 (1.05 – 1.48)	2.20 (1.62 – 3.00)	1.54 (1.24 – 1.90)	0.86 (0.68 – 1.04)
Mn ($x 10^{-4}$)	3.72 (2.77 – 4.90)	7.98 (5.37 – 11.6)	5.40 (4.21 – 7.19)	3.54 (2.50 – 4.62)
Fe ($x 10^{-3}$)	4.37 (3.74 – 5.27)	10.3 (6.01 – 16.2)	6.61 (4.97 – 8.82)	4.04 (2.84 – 6.69)
Co ($x 10^{-3}$)	2.01 (1.70 – 2.28)	1.62 (1.03 – 2.19)	1.83 (1.48 – 2.21)	0.55 (0.27 – 0.89)
Ni ($x 10^{-3}$)	0.79 (0.63 – 0.98)	1.25 (0.82 – 1.81)	1.02 (0.79 – 1.33)	0.48 (0.35 – 0.62)
Cu ($x 10^{-4}$)	2.73 (1.10 – 5.55)	5.20 (1.87 – 9.32)	4.07 (1.65 – 6.49)	1.76 (0.72 – 3.14)
Zn	0.85 (0.76 – 0.89)	0.74 (0.43 – 0.88)	0.81 (0.67 – 0.89)	0.21 (0.09 – 0.33)
Ca:Zn	0.16 (0.11 – 0.31)	0.32 (0.11 – 1.27)	0.22 (0.11 – 0.47)	3.54 (1.98 – 10.14)

Supplementary Table S5. Comparison of calcification clusters (2 – 4) using Kruskal-Wallis tests, with a Dunn-Sidak post-hoc correction for multiple comparisons. All quoted *p* values are Dunn-Sidak corrected values unless otherwise stated. All significant values are highlighted (Red: $p<0.05$, Yellow: $p<0.01$, Green: $p<0.001$).

Ca ratio

	2	3	4
3	<0.001		
4	<0.001	<0.001	
5	<0.001	<0.001	<0.001

Ti ratio

	2	3	4
3	0.010		
4	0.858	<0.001	
5	0.899	<0.001	0.109

Cr ratio

	2	3	4
3	<0.001		
4	0.797	<0.001	
5	0.715	<0.001	0.019

Mn ratio

	2	3	4
3	<0.001		
4	0.476	0.002	
5	0.986	<0.001	0.052

Fe ratio

	2	3	4
3	<0.001		
4	0.303	0.002	
5	0.511	0.004	0.998

Co ratio

	2	3	4
3	0.038		
4	0.832	0.065	
5	<0.001	<0.001	<0.001

Ni ratio

	2	3	4
3	0.053		
4	0.535	0.498	
5	0.570	<0.001	<0.001

Cu ratio

	2	3	4
3	0.461		
4	0.980	0.481	
5	0.799	<0.001	0.187

Zn ratio

	2	3	4
3	<0.001		
4	0.925	<0.001	
5	<0.001	<0.001	<0.001

Ca: Zn ratio

	2	3	4
3	<0.0010		
4	0.093	<0.001	
5	<0.001	<0.001	<0.001

Supplementary Table S6. Median calcification area and elemental ratios for each cluster type (A – E) with lower and upper quartiles in brackets. Kendall's tau (r_τ) statistics and related *p* values for each parameter.

	Type					r_τ	p
	A	B	C	D	E		
Calc Area /µm^2	2500 (1600 – 8100)	24600 (7500 – 115600)	12500 (4175 – 22025)	3750 (2350 – 15200)	45800 (12400 – 111650)	0.40	**<0.001**
Ca	0.88 (0.71 – 0.96)	0.51 (0.41 – 0.72)	0.48 (0.40 – 0.55)	0.24 (0.11 – 0.33)	0.11 (0.09 – 0.17)	-0.321	**<0.001**
Ti (x 10^{-3})	1.22 (0.95 – 1.32)	1.15 (1.03 – 1.22)	1.17 (1.16 – 1.39)	1.68 (1.46 – 2.05)	1.51 (1.35 – 1.56)	-0.048	0.617
Cr (x 10^{-3})	1.01 (0.87 – 1.31)	1.29 (1.07 – 1.54)	1.28 (1.20 – 1.48)	2.10 (1.50 – 2.54)	1.69 (1.63 – 1.84)	0.280	**<0.001**
Mn (x 10^{-4})	4.75 (3.76 – 5.47)	4.47 (3.96 – 5.04)	5.04 (4.53 – 5.75)	8.50 (6.34 – 10.2)	6.23 (5.41 – 9.13)	0.011	0.911
Fe (x 10^{-3})	5.26 (3.97 – 17.9)	5.83 (3.90 – 6.10)	4.02 (0.99 – 1.36)	11.4 (7.10 – 20.6)	6.81 (4.51 – 11.9)	0.077	0.416
Co (x 10^{-3})	0.35 (0.21 – 0.70)	1.14 (0.66 – 1.40)	1.23 (0.99 – 1.36)	1.87 (1.53 – 2.05)	2.08 (1.95 – 2.27)	0.307	**<0.001**
Ni (x 10^{-3})	0.58 (0.51 – 0.73)	0.77 (0.64 – 0.87)	0.82 (0.70 – 1.09)	1.36 (0.83 – 1.83)	1.19 (0.97 – 1.21)	0.287	**<0.001**
Cu (x 10^{-4})	2.66 (1.87 – 3.87)	2.68 (2.01 – 3.49)	3.34 (2.18 – 3.97)	5.66 (3.85 – 6.79)	4.65 (4.03 – 6.16)	0.015	0.875
Zn	0.05 (0.02 – 0.27)	0.48 (0.25 – 0.58)	0.51 (0.44 – 0.59)	0.72 (0.65 – 0.86)	0.88 (0.81 – 0.89)	0.350	**<0.001**
Ca:Zn	18.2 (2.64 – 45.7)	1.11 (0.70 – 2.88)	0.94 (0.68 – 1.25)	0.35 (0.13 – 0.51)	0.12 (0.10 – 0.21)	-0.136	0.190

Significant values (p<0.05) are highlighted in **bold**.

Supplementary Table S7. Comparison of calcification types (A – E) using Kruskal-Wallis tests, with a Dunn-Sidak post-hoc correction for multiple comparisons. All quoted *p* values are Dunn-Sidak corrected values unless otherwise stated. All significant values are highlighted (Red: $p<0.05$, Yellow: $p<0.01$, Green: $p<0.001$).

Ca ratio

	A	B	C	D
B	0.273			
C	0.033	0.993		
D	<0.001	0.647	0.925	
E	<0.001	0.133	0.380	0.941

Ti ratio

	A	B	C	D
B	0.973			
C	1.000	0.997		
D	0.039	0.043	0.119	
E	0.094	0.098	0.247	0.995

Cr ratio

	A	B	C	D
B	0.889			
C	0.759	1.000		
D	<0.001	0.251	0.288	
E	<0.001	0.287	0.328	1.000

Mn ratio

	A	B	C	D
B	0.999			
C	0.999	0.993		
D	0.019	0.069	0.224	
E	0.109	0.242	0.574	0.971

Fe ratio

	A	B	C	D
B	1.000			
C	0.766	0.971		
D	0.373	0.493	0.081	
E	0.959	0.946	0.437	0.898

Co ratio

	A	B	C	D
B	0.423			
C	0.164	1.000		
D	<0.001	0.576	0.745	
E	<0.001	0.095	0.163	0.933

Ni ratio

	A	B	C	D
B	0.642			
C	0.112	0.980		
D	<0.001	0.314	0.744	
E	<0.001	0.361	0.815	1.000

Cu ratio

	A	B	C	D
B	1.000			
C	0.999	0.995		
D	0.127	0.271	0.555	
E	0.053	0.194	0.455	1.000

Zn ratio

	A	B	C	D
B	0.255			
C	0.024	0.989		
D	<0.001	0.678	0.955	
E	<0.001	0.125	0.405	0.915

Ca:Zn ratio

	A	B	C	D
B	0.260			
C	0.027	0.991		
D	<0.001	0.657	0.940	
E	<0.001	0.138	0.415	0.941

Calcification Area

	A	B	C	D
B	0.139			
C	0.495	0.986		
D	0.984	0.617	0.939	
E	0.009	1.000	0.916	0.296

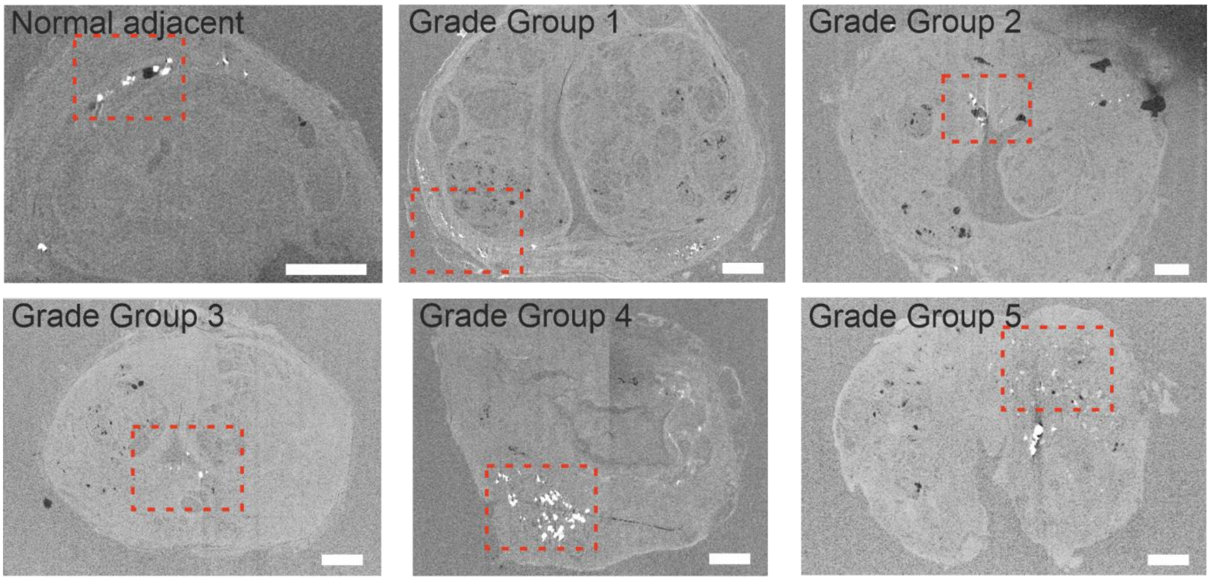

Supplementary Figure S2. Example μCT images of prostate megablocks from each tissue Grade Group, with regions of interest marked with a red box. Samples match the calcification images in Figure 2. Scale bar = 5 mm.

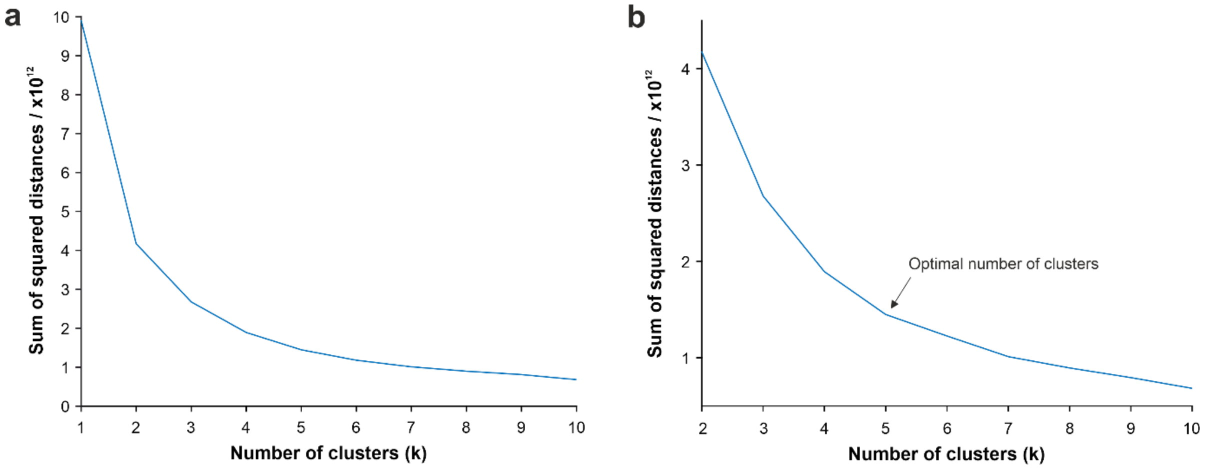

Supplementary Figure S3. Elbow curve using sum of squared distances plot. a) Plot for k = 1 to k = 10 clusters. Separation of clusters 1 and 2 was highly affected by the surrounding soft tissue, suggesting that two clusters may be the optimal number. However, in order to also cluster within calcifications, k must be greater than 2. b) Plot for k = 2 to k = 10 clusters, better demonstrating the optimal number of calcification clusters. Five clusters were chosen as the optimal number of clusters using the elbow method.
